# Hybrid Nerve Sheath Tumor Detected by Endoscopic Full‐Thickness Resection for a Gastric Subepithelial Lesion: A Case Report

**DOI:** 10.1002/deo2.70276

**Published:** 2026-01-13

**Authors:** Mai Fukuda, Masakuni Kobayashi, Miku Maeda, Mamoru Ito, Naoya Tada, Toshiki Futakuchi, Naoto Tamai, Nei Fukasawa, Masayuki Shimoda, Kazuki Sumiyama

**Affiliations:** ^1^ Department of Endoscopy The Jikei University School of Medicine Tokyo Japan; ^2^ Digestive Diseases Center, Showa Medical University Koto Toyosu Hospital Tokyo Japan; ^3^ Department of Pathology The Jikei University School of Medicine Tokyo Japan

**Keywords:** endoscopic full‐thickness resection, endoscopic ultrasound, gastric subepithelial lesion, hybrid nerve sheath tumor, hybrid schwannoma–perineurioma

## Abstract

Hybrid nerve sheath tumors (HNSTs) are exceedingly rare in the gastrointestinal tract, particularly in the stomach. We describe a case of an enlarging gastric subepithelial lesion (SEL) that was accurately diagnosed and curatively treated by endoscopic full‐thickness resection (EFTR). A 50‐year‐old woman presented with a 10 mm SEL on the posterior wall of the upper gastric curvature. Endoscopic ultrasound (EUS) revealed a low‐hypoechoic lesion primarily originating from the third layer with focal, indistinct borders with the muscularis propria. Initial boring biopsy suggested a granular cell tumor based on morphology and SOX10/S100 positivity. Six months later, the lesion had enlarged to 15 mm, and EFTR under general anesthesia with laparoscopic backup was selected to obtain a full‐thickness specimen. En bloc resection was successfully achieved, and the defect was completely closed with clips. Histopathological and immunohistochemical examinations revealed biphasic Schwann and perineurial differentiation, confirming a hybrid schwannoma/perineurioma. The postoperative course was uneventful, and no recurrence was observed during the 22‐month follow‐up. This case highlights the diagnostic value of EFTR for rare neurogenic SELs in which superficial biopsy may be inconclusive.

## 1 Introduction

Hybrid nerve sheath tumors (HNSTs) are rare benign peripheral NSTs composed of at least two components, most commonly schwannoma and perineurioma [1]. Although they typically arise in superficial soft tissues, gastrointestinal involvement is extremely uncommon [2, 3]. Accurate diagnosis requires adequate tissue for morphological and immunohistochemical evaluation, which is often difficult when subepithelial lesions (SELs) originate from or involve the muscularis propria. Traditionally, such lesions require surgical resection, but endoscopic full‐thickness resection (EFTR) has recently emerged as a minimally invasive alternative that enables both en bloc removal and definitive pathological diagnosis. We report a rare gastric HNST that was successfully managed with EFTR.

## 2 Case Report

**FIGURE 1:**
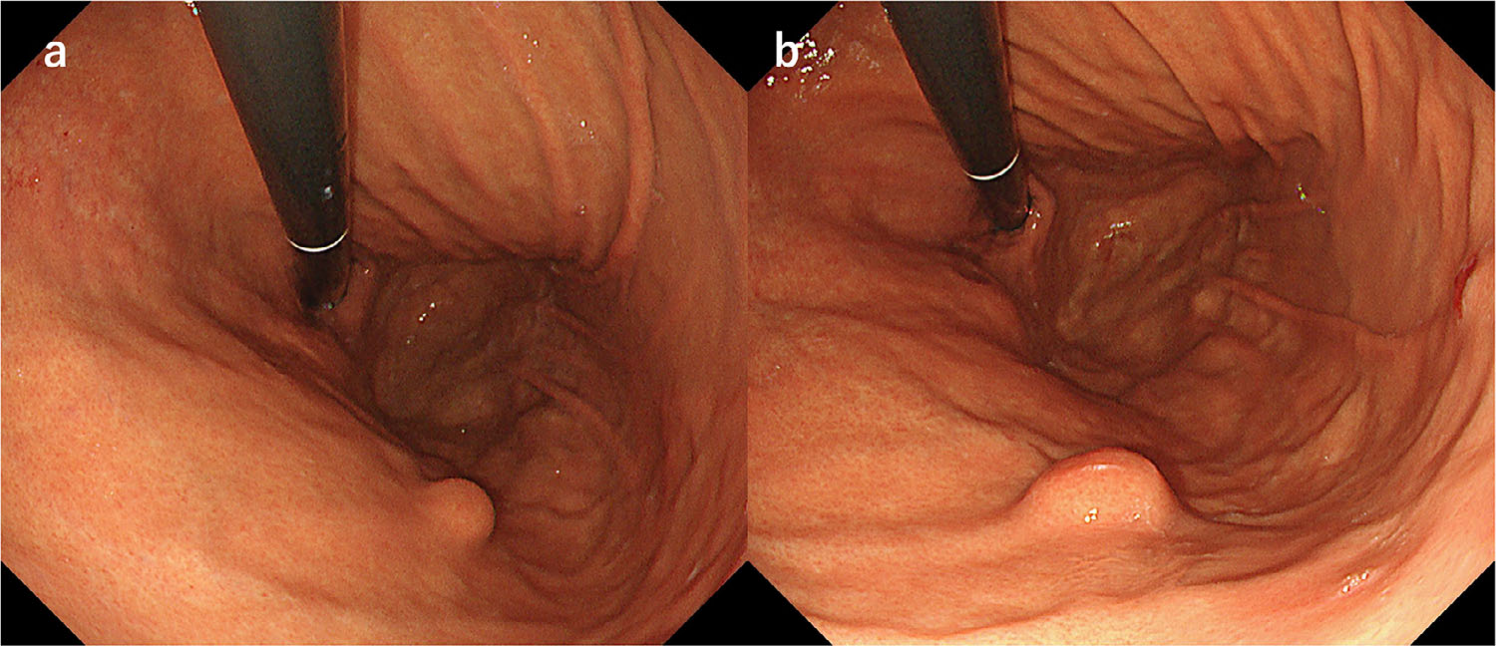
Endoscopic and endosonographic imaging of the lesion. (a) A subepithelial lesion (SEL) is visualized on the posterior wall of the upper gastric curvature. (b) The subepithelial lesion (SEL) shows enlargement at the 6‐month follow‐up. The SEL appears on the fourth layer of the gastric wall in the endoscopic ultrasound image, measuring 8.4 mm in diameter.

**FIGURE 2:**
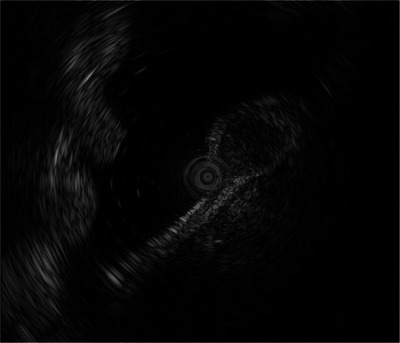
The lesion was mainly situated within the third layer (hyperechoic, corresponding to the submucosa), with partially indistinct borders with the fourth layer (hypoechoic, muscularis propria).

A 50‐year‐old woman was found to have a 10 mm SEL on the posterior wall of the upper gastric curvature (Figure 1a). EUS showed the characteristic five‐layer structure with a low‐hypoechoic lesion primarily arising from the third layer but with focal indistinct margins with the muscularis propria (Figure 2). Initial boring biopsy indicated a granular cell tumor. Six months later, the lesion had enlarged to 15 mm (Figure 1b), and endoscopic resection was planned. As muscularis involvement could not be excluded and a full‐thickness specimen was considered necessary for diagnosis, EFTR was selected. The procedure was performed under general anesthesia with laparoscopic and surgical backup present.

After a circumferential mucosal incision, a multi‐loop traction device (MLTD) with a Sure Clip (Micro‐Tech Co., Ltd., Nanjing, China) was applied to the oral side, with counter‐traction placed on the opposite side to pull the lesion toward the lesser curvature. This bidirectional traction provided stable exposure of the muscularis propria, enabling optimal visualization for intentional perforation and controlled full‐thickness dissection, resulting in en bloc resection. The defect, including the perforation site, was then completely closed using MANTIS (Boston Scientific, Marlborough, MA, USA) and additional SureClips, with pre‐placed clips facilitating smooth and efficient longitudinal closure (Details of the procedure are presented in Video S1).

The postoperative course was uneventful, and the patient was discharged on postoperative day 7. No recurrence was detected during the 22‐month follow‐up via endoscopy and abdominal computed tomography.

## 3 Pathological Findings

**FIGURE 3:**
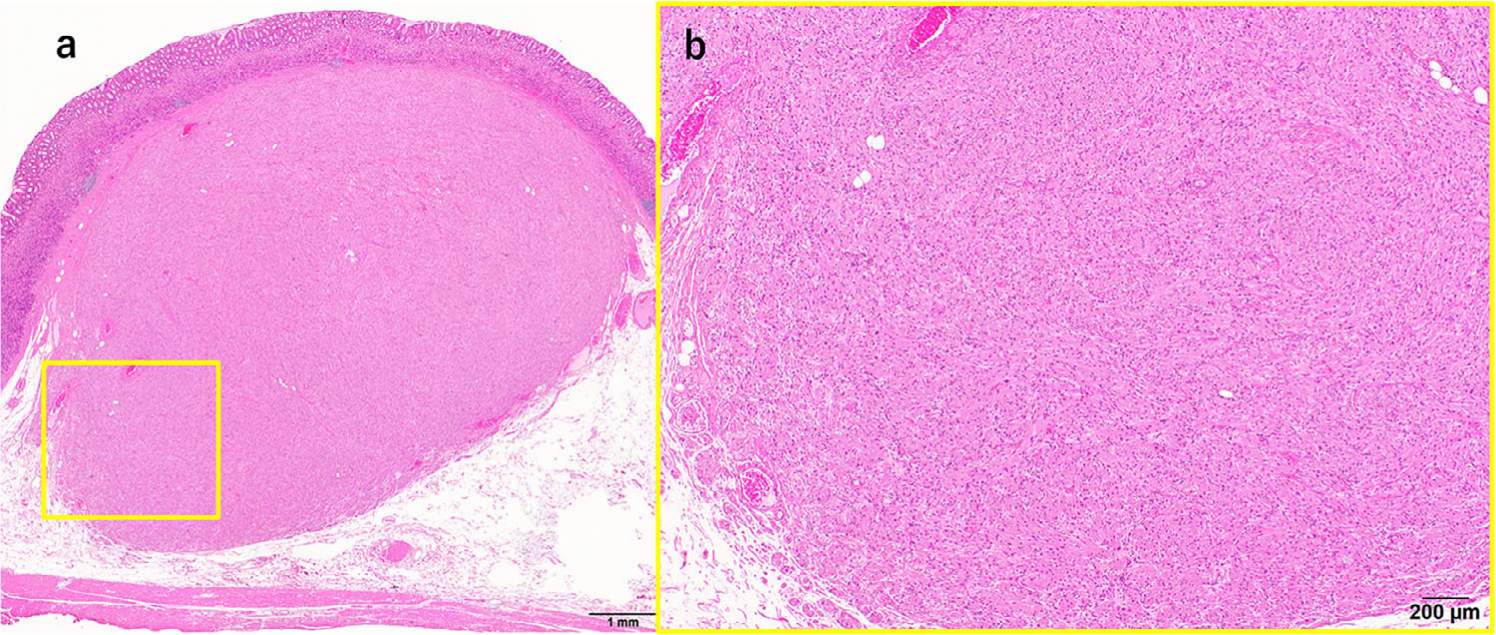
(a) Histopathological findings of the resected specimen. Hematoxylin and eosin staining. A low‐power view reveals a well‐circumscribed, unencapsulated nodular lesion predominantly situated within the submucosa. (b) Low magnification sections demonstrate a biphasic architecture with Schwann‐like epithelioid to spindle areas intermingled with perineurial‐like fascicles.

Gross examination revealed a well‐circumscribed, unencapsulated 12 × 11 × 8‐mm^3^ nodule in the submucosa with slight extension into the lamina propria (Figure 3a). Microscopically, two intimately admixed components were identified. One consisted of large epithelioid to spindle cells with granular cytoplasm arranged in nests, and the other showed fascicular spindle cells with perineurial morphology (Figure 3b).

**FIGURE 4:**
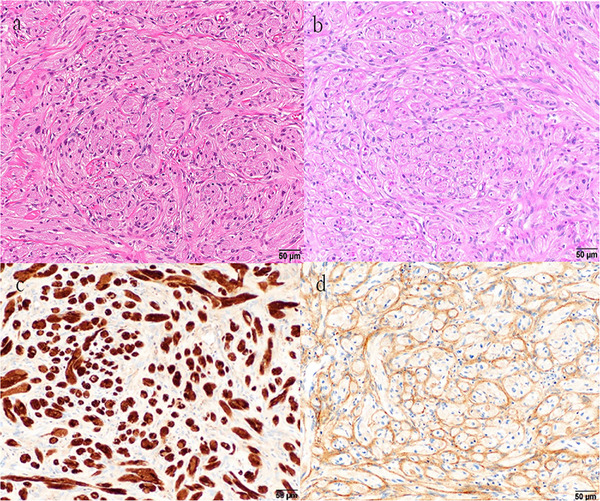
Histopathological and immunohistochemical findings of the resected specimen. (a) Hematoxylin and eosin staining, at higher magnification. The tumor comprises two intermingled components: nests of large epithelioid cells with abundant eosinophilic granular cytoplasm, surrounded by a fascicular and whorled proliferation of spindle cells with wavy nuclei. (b) Periodic acid‐Schiff stain. Some of the large epithelioid cells demonstrate partially positive intracytoplasmic granules. (c) S100. The large epithelioid cells exhibit diffuse positivity. (d) EMA. The spindle‐shaped cells are immunopositive.

Silver impregnation demonstrated pericellular reticular fibers around epithelioid cells, and PAS staining revealed focal intracytoplasmic granules. Schwann‐like cells were diffusely positive for S100 and SOX10, while perineurial cells stained for EMA, Claudin‐1, and GLUT‐1 (Figure 4a–d). The Ki‐67 index was <1%, and H3K27me3 expression was retained. The tumor was negative for DOG‐1 and CD117. These findings supported the diagnosis of a hybrid schwannoma/perineurioma, which was completely resected with negative margins.

## 4 Discussion

HNSTs are rare neoplasms composed of elements from more than one peripheral NST subtype. Only isolated cases have been described in the stomach [4, 5]. Histologically, they display an intimate admixture of Schwann and perineurial areas, necessitating immunohistochemistry for accurate classification. Complete resection is usually curative, as malignant transformation or recurrence is exceptional, and Ki‐67 indices remain low [6].

In this case, the initial biopsy mimicked a granular cell tumor, but the subsequent full‐thickness specimen revealed clear biphasic architecture and distinct immune profiles. Differentiating GCT from schwannoma is notoriously challenging because both are derived from Schwann cells. However, the absence of calretinin and the pattern of reticular fibers favored a schwannian component. Perineurial cells were confirmed using EMA and GLUT‐1 positivity. Thus, the lesion was best classified as a hybrid schwannoma/perineurioma type of HNST.

Conventionally, gastric SELs involving the muscularis propria required surgical resection for treatment and diagnosis. EFTR has recently emerged as a minimally invasive alternative offering en bloc resection and definitive histology without gastrectomy. The present case demonstrated that EFTR can safely achieve R0 resection with precise pathological characterization of rare lesions. The technique requires experience in managing intentional perforation and achieving secure closure, but advances in devices such as traction systems and pressure‐control insufflators have enhanced its safety profile [7, 8].

This case also underscores EFTR's diagnostic value for rare neurogenic tumors. Superficial biopsies may yield misleading results because of limited sampling depth. In contrast, a full‐thickness specimen allows recognition of dual nerve sheath differentiation and exclusion of gastrointestinal stromal tumor or smooth‐muscle tumors. Integrating EFTR with detailed immunohistochemistry and future molecular studies could refine the classification and management of such tumors.

As EFTR gains acceptance, more cases of rare entities such as HNSTs are likely to be identified, broadening our understanding of their clinicopathological spectrum. Further case accumulation and genetic analyses are needed to clarify their pathogenesis and behavior. This report adds to the limited literature on gastric HNSTs and highlights the potential of EFTR as a diagnostic and therapeutic modality for rare SELs.

## Author Contributions

**Mai Fukuda**: review and editing (equal); **Masakuni Kobayashi**: review and editing (equal); **Miku Maeda**: review and editing (equal); **Naoya Tada**: review and editing (equal); **Mamoru Ito**: review and editing (equal); **NEI Fukasawa**: review and editing (equal); **Toshiki Futakuchi**: review and editing (equal); **Naoto Tamai**: review and editing (equal); **Masayuki Shimoda**: review and editing (equal); **Kazuki Sumiyama**: review and editing (equal).

## Conflicts of Interest

Kazuki Sumiyama is a deputy editor‐in‐chief of DEN Open. The other authors declare no conflicts of interest.

## Funding

The authors have nothing to report.

## Ethics Statement

**Approval of the research protocol by an Institutional Reviewer Board**: N/A.

## Consent

N/A.

## Clinical Trial Registration

N/A.

## Supporting information



Supporting Video
